# Chronic Tumour in the Abdomen, Produced by a Large Accumulation in the Superior Portion of the Colon, &c.

**Published:** 1831

**Authors:** 


### XXIX.

### Chronic Tumour in the Abdomen, produced by a large Accumulation in the Superior Portion of the Colon, &c. By John Howship, Esq.

July, 1830, I had the opportunity of examining the body of a tall, stout woman, aged 72, who after many months confinement to bed had died, with the usual symptoms of disease in the uterus.

Upon enquiry of those who had attended upon and nursed the patient, I understand, as it appeared that the ascertained that she had complained of constant pain in the bowels for the last twelve months, with a sense of swelling, or tumor, within the abdomen; which swelling was very large, and, as the nurse said (who had frequently examined it), felt uniformly soft and yielding, like a sponge, being principally situated in the right side; although, in changing the position in bed, she had found it, like a great weight, fall from side to side. If, for instance, she lay on the right side, a part of it seemed to roll over to that side; and if she turned over to the left side, she immediately felt the heavy mass fall over to that side, with a dragging sensation hardly to be borne.

The frequent magnitude of this tumor within the abdomen was said to have equalled, or even exceeded, that of a pregnant woman at her full time; and this remark the patient herself had repeatedly made. After death, the existence of tumor in the right hypogastrium was sufficiently manifest to the eye; it now felt soft, flaccid, and pulpy.

Laying open the cavity of the abdomen, I found this tumor was caused by a considerable enlargement of the whole of the colon, but particularly, as it appeared, of the cœcum and adjacent part of the great intestine. The peritoneal covering also, of these parts of the intestine, was so relaxed and elongated, that the weight of the parts, when the body was laid on the left side, brought the cœcum down to the middle of the abdomen.

Passing a ligature round the middle of the transverse arch, I removed the cœcum and superior portion of the colon, washed out the contents—a very large quantity of stiff and pulpy, yet well-digested, fæculent matter—and then tying the lower opening, inflated the intestine from the ileum.

In this operation I was repeatedly struck by observing, that although the air passed down freely enough into the cœcum and colon, not any appeared to escape back again into the small intestine. This circumstance I could the less understand, as it appeared that the portion of gut, where the cœcum is parted off from the colon, was so enlarged, that the valve there situated was in all probability useless. The inflation, however, was completed, the ileum tied up, and the preparation hung up to dry.

The next day, expecting to see it dry and expanded, I found it damp, flaccid, and collapsed. The tied end of the ileum was therefore cut off, the intestine moistened in warm water, and again inflated, when it seemed that the air escaped, although I could not discover where, till Dr. P. Robertson, who was with me at the time, suggested the placing it under water; we then perceived the bubbles of air rise from a minute ulcerated opening in the cœcum, which, neither thickened nor discoloured, would otherwise have escaped detection. This secured by a ligature, the intestine retained the air, and was soon dry.

On subsequently cutting an opening into the cœcum, I was so fortunate as to find that the peculiar and very curious state of the parts was well demonstrated.

The original valve of the cœcum, as I had anticipated, was so expanded and drawn aside, by the progressive enlargement of the cavity of the gut, as to have been, for a long time, entirely inefficient; and the consequence was, that Nature, ever watchful over the movements of the animal machine, had contrived (the first valve failing) to form a second, by the readiest and most ingenious device imaginable—that of simply drawing the inner membrane of the gut across the termination of the ileum, in the cœcum.

The dried preparation, which is preserved in my collection, demonstrates the enlargement of the cœcum and colon, the expanded figure of the original valve of the cœcum, and the position and appearance of the new valve closing the opening from the ileum; an opaque middle line marking the junction of the two portions of the valve.

*Observations.*—In some practical observations on the diseases of the lower bowels, published several years since, I endeavoured to state clearly what I had seen, in proof that permanent lodgments occasionally take place within the cells of the colon, notwithstanding the apparently free operation of active purgatives. This fact I was desirous to state clearly, because it is a point upon which I had myself been extremely sceptical; but the proofs there adduced are, I apprehend, so conclusive, as to render any further evidence of the fact unnecessary.

In the present case, however, the condition of the intestinal canal was such as was altogether new to me, and such as I could not have anticipated. The colon presented no cells or recesses, nor any portion of the great intestine a point of contraction, either from disease or permanent spasm; the whole of the colon and rectum being found, on examination, perfectly free, and moderately occupied. In fact, although the usual habits of the bowels had been those of confinement, for the last fortnight of the patient's life, they had been constantly and copiously relaxed; and notwithstanding this relaxation, the same large tumor appeared to remain, which had for a twelvemonth before induced various feelings of uneasiness, and frequently those of great distress; and this, notwithstanding there must at all times have been a free way through the midst of the mass so often as the bowels were relieved, either by spontaneous action, or the operation of medicine.—*Medical Gazette.*

We have had very numerous opportunities of witnessing collections in the caput coli and ascending arch of the colon, where the symptoms and the manual examinations led to the inference that the liver was enlarged. It is not many weeks since we were led into this error, after all former experience. The exhibition of opiates, conjoined or alternated with purgatives, throws light on these complaints, though it sometimes requires weeks of treatment before the accumulations are broken up and evacuated.—*Ed.*

